# A unified framework for van ‘t Hoff’s law: addressing the complexity of osmotic concentration

**DOI:** 10.1098/rsos.250622

**Published:** 2025-09-15

**Authors:** Serena Y. Kuang, Xiaonan Li, Xiaoqi Yang, Eric Jones

**Affiliations:** ^1^ Foundational Medical Studies, Oakland University William Beaumont School of Medicine, Rochester, MI, USA; ^2^ Children Healthcare, Children Hospital of Nanjing Medical University, Nanjing, Jiangsu, People’s Republic of China; ^3^ Icahn School of Medicine at Mount Sinai, New York, USA

**Keywords:** osmosis system, osmotic concentration, osmotic pressure, membrane-dependence, van 't Hoff's law, osmotic coefficient, reflection coefficient, biological membrane

## Abstract

The original van ‘t Hoff’s law established the theoretical foundation for osmosis but applies only to ideal solutions and membranes. To address real-world complexities (non-ideal solutions, diverse membranes, etc.), multiple variations have emerged over a century. In resolving osmosis-related conceptual issues, our previous work introduced several new fundamental concepts to fill gaps in the study of osmosis and redefined osmotic concentration (OC) as a membrane-dependent, osmosis system-level parameter, not a parameter of any isolated solution. This article examines the multiple factors influencing the initial OC (OC_0_) before osmosis occurs and demonstrates that the multiple forms of van ‘t Hoff’s law can be unified using OC_0_ into one general form through mathematical reasoning. Building upon this unified framework, we further propose an extended formulation to accommodate more complex osmosis systems. These general forms of van ‘t Hoff's law overcome the limitations of the original and may be widely applied to real-world dilute solutions and membranes. We also perform an initial validation of our work using measured data in the literature. This work represents a significant theoretical advance in the understanding of osmosis and has potential to impact multiple disciplines that teach and research it, including physics, chemistry, physiology and clinical disciplines.

## 1. Introduction

The phenomenon of osmosis was first documented by the French scientist Jean-Antoine Nollet in 1748 [1], and the term ‘osmosis’ was first introduced by French physiologist René Dutrochet in 1826 [2]. The osmotic pressure of solutions was first accurately measured by the German botanist Wilhelm Pfeffer in 1877 [3], using a device he invented called the Pfeffer Cell. The theoretical foundation of osmosis was established by Dutch chemist Jacobus H. van ‘t Hoff, who applied ideal gas laws to dilute solutions in his Nobel Prize-winning work [4], formulating the original form of van ‘t Hoff’s law: *π* = *C⋅RT*, where *π* is the osmotic pressure, *R* is the gas constant, *T* is the absolute temperature and *C* is the molar concentration of solutes [5,6]. Since then, over 100 years have passed, and the original van ‘t Hoff’s law has evolved into multiple forms to accommodate different types of solutions and semipermeable membranes.

Osmosis is now widely studied and taught across many disciplines, including physics, chemistry, biology, physiology and numerous clinical sciences (e.g. internal medicine, surgery, anaesthesiology, endocrinology, nephrology, paediatrics, etc.). However, owing to the absence of several fundamental concepts, the definition of osmolarity (osmotic concentration, OC) has been imprecise, leading to the coexistence of two types of osmolarity [7–9]: osmolarity (the concentration of total solute particles (TSP) in a solution) and effective osmolarity (the concentration of impermeant solute particles (SP) facing a semipermeable membrane). To address these issues, our previous work introduced the missing fundamental concepts and, based on this foundation, redefined osmolarity through logical reasoning [10] to eliminate the confusion caused by the coexistence of the two forms [11]. During this process, we unexpectedly realized that the redefined osmolarity concept unifies the multiple forms of van ‘t Hoff’s law into one general form. This unification not only confirms the appropriateness of the redefinition but also advances the theoretical foundation of osmosis. This outcome was published in abstract form [12] with mathematical details of the reasoning process summarized in a table.

This article aims to illustrate the complexity of the redefined osmolarity concept and significantly expand the published abstract [12] to demonstrate how the concept unifies the multiple forms of van ‘t Hoff’s law. Building on this foundation, we further propose a more generalized formulation of van ’t Hoff’s law to accommodate complex osmosis systems that the redefined osmolarity fails to address. To reach this goal, in the Introduction, we summarize the four fundamental concepts we introduced previously and the redefined osmolarity concept (simply, osmolarity) [10], address the limitations of the original form of van ‘t Hoff’s law and highlight two macroscopic expressions of the driving force behind osmotic phenomena.

### 1.1. Past key contributions to the study of osmosis

A total of five key concepts are summarized: the four fundamental concepts as the prerequisites for studying osmosis and the new definition of osmolarity [10]:

(i) concept 1: a simple osmosis system with a solution compartment (S) and a water compartment (H_2_O) separated by a selectively permeable membrane (m) denoted as S-m-H_2_O;
(ii) concept 2: a composite osmosis system denoted as S_1_-m-S_2_, which can be deconstructed into two simple osmosis systems that are mirrored (i.e. S_1_-m-H_2_O and H_2_O-m-S_2_);
(iii) concept 3: **C_TSP_**, the molar concentration of the TSP in a solution measured using mM, not mOsm L^−1^. Because this concept is absent, the conventional concept of osmolarity mistakes its position, but using mOsm L^−1^ as its unit. In other words, **C_TSP_** has long been regarded as conventional osmolarity. **C_TSP_** is an inherent property of a solution and serves as the source of osmolarity. It should be **C_TSP_** that informs the colligative properties of a solution, not osmolarity;
(iv) concept 4: membrane (m)-dependency of all osmosis-related concepts. Osmosis-related concepts include osmoles, osmotically active SP, osmolarity, osmolality, osmotic pressure, osmotic pressure gradient and tonicity. These are all related to impermeant SP and exclude permeant SP—an explicit and systematic approach to all osmosis-related concepts; and
(v) concept 5: definition of osmolarity in terms of a simple S-m-H_2_O. When a solution becomes a part of S-m-H_2_O, its **C**_**TSP**_ interact with the given m and differentiate into a permeant fraction and an impermeant fraction, which are both m-dependent. The impermeant fraction of **C**_**TSP**_ in a simple S-m-H_2_O is the m-dependent osmolarity, which is a variable during osmosis. Osmolarity is OC. Its initial value, OC_0_ (*t* = 0) before osmosis occurs, is of practical use. When m is ideal (only permeable to water), the impermeant fraction of **C**_**TSP**_ is 100%.

These five key concepts establish the new theoretical principles for the study of osmosis. Since the International Union of Pure and Applied Chemistry (IUPAC) officially discontinued the term ‘osmolarity’ and replaced it with ‘OC’ in 1996 [13], OC_0_ is used throughout this article. The word ‘osmolarity’ may be used occasionally depending on the context.

### 1.2. Limitations of the original form of van ‘t Hoff’s law

When using the original form of van ‘t Hoff’s law, the following criteria need to be met [14]:

(i) the solution contains a single type of non-dissociable molecule, such as glucose molecules;
(ii) the solution is sufficiently dilute to neglect interactions between SP that could reduce their osmotic effect. Such a solution is commonly referred to as ideal; and
(iii) the membrane is ideal, i.e. permeable to water only, not permeable to any SP species.

The first and third assumptions are very unrealistic when dealing with biological systems. If the composition of the solution becomes more complex (such as a mixture of glucose, NaCl and MgSO_4_), or if the membrane has some permeability to SP, or if the non-ideality of a solution cannot be ignored, alternate forms of van ‘t Hoff’s law have been derived to adapt to these situations. However, what constitutes OC_0_ in these derivative forms is unclear^1^ if OC_0_ in a simple S-m-H_2_O is not defined.

In terms of the second criterion, body fluids (extracellular and intracellular fluids) are considered dilute solutions because they are primarily composed of water (>95%) and have relatively low solute concentrations [16], but they are not ideal solutions [17]. Their non-ideality is often taken into account when conventional osmolarity (actually the **C_TSP_**) is calculated in physiology or clinical practice.

These limitations inform the sources of the complexity of OC_0_. The factors that determine the value of OC_0_ originate from the solution and/or the membrane. The solution contributes two sources of complexity: possible diverse SP composition and possible non-ideality if it is not sufficiently dilute. Both artificial and biological membranes can be extremely variable in terms of their physical, chemical and biological properties. They can differ in thickness and in the number, types and sizes of pores, and the transport properties of pores (such as transport proteins or ion channels) in biological membranes are often not constant but regulated by physiological factors, such as hormones.

For these reasons, OC_0_, which results from the interaction between a complex solution and a complex membrane, is itself rather complex. This is why, historically, multiple forms of van ‘t Hoff’s law have been developed over time to adapt to the complexity of solutions and membranes. These many forms of van ‘t Hoff’s law are analysed in §2. Owing to the influence of many contributing factors, OC_0_ is, in essence, an *effective* initial OC. However, it differs from the concept of effective osmolarity [11] previously mentioned. In physiology, effective osmolarity is a property of a solution, when the concept of a simple osmosis system has not yet been introduced. By contrast, OC_0_ is defined in the context of a simple osmosis system, and therefore it is a property of the system, not of the solution.

### 1.3. Two macroscopic expressions of the driving force behind osmotic phenomena

In physics and chemistry, osmosis in a simple S-m-H_2_O is commonly described as being driven by the difference in the chemical potential of water between two compartments. From this perspective, water flows from the side with higher water chemical potential to the side with lower water chemical potential. This gradient, denoted as Δ*μ*_₀_, serves as the macroscopic driving force of osmosis, and the osmotic pressure *π* = const·Δ*μ*_₀_, where Δ*μ*_₀_ pushes water to the S compartment, and ‘const’ is the abbreviation of a constant (physical and chemical perspective) [18]. By contrast, in the biomedical fields, it is common to say that solutes ‘attract’ water, or that water follows salts. That is, osmosis in the same S-m-H_2_O is often described as water moving from the water compartment towards the solution compartment (S) and osmotic pressure is described using van 't Hoff’s law: *π* = *RT·C*. These two views (i.e. Δ*μ*_₀_-driven water flow versus solutes ‘attracting’ water) are in fact two sides of the same coin. Both describe the macroscopic consequence of an uneven distribution of SP and solvent (water) molecules across a semipermeable membrane.

Because the present paper focuses on van ‘t Hoff’s law, where solute concentration serves as the independent variable and osmotic pressure as the dependent variable, we adopt the biomedical convention: namely, that solutes ‘attract’ water. Therefore, osmosis in a composite S_1_-m-S_2_ is considered a water competition ‘game’ between solutes in the two solution compartments (S_1_ and S_2_). However, it is important to clarify that solutes do not possess an intrinsic ability to ‘pull’ or ‘attract’ water. This phrase is merely a convenient macroscopic description of a process that appears to behave this way. The underlying microscopic mechanism for generating the driving force of osmosis stems from molecular thermodynamic processes involving interactions among water molecules, impermeant SP and the membrane [19,20]. Understanding this mechanism requires a background in physics and considerable explanation, which is beyond the scope of this article. On the other hand, it is true that under steady-state conditions, with complete solute impermeability, the membrane may be treated as a black box, as is commonly done in irreversible thermodynamics. In such cases, only the water chemical potential difference or the solute concentration difference across the membrane matters, and membrane-specific details are ignored.

## 2. Results and discussion

In this section, we present the multiple forms of van ‘t Hoff’s law one by one and represent each using the general form of *π* = *RT·OC*_0_ through logical reasoning. Depending on the reference, a given physical parameter in van ‘t Hoff’s law may be represented by different letters. To simplify comparison and analysis during our logical reasoning, we have modified the various forms of the law when necessary to ensure that identical parameters are represented by identical symbols. We further assume that all dissociable molecules are completely dissociated:

(i) ‘*g*’ denotes the number of dissociable particles per solute molecule. For example, *g*_glu_ = 1, *g*_NaCl_ = 2, and *g*_CaCl2_ = 3;
(ii) *C*_*i*_ denotes the molar concentration of a SP species i (e.g. *C*_glu_, *C*_Na_, *C*_Cl_, etc.); and
(iii) *C*_*j*_ denotes the molar concentration of a type of solute molecule *j* (e.g. *C*_glu_, *C*_NaCl_, *C*_MgSO4_, etc.) regardless of whether the type of molecule is dissociable or non-dissociable. For a type of non-dissociable molecule such as glucose, *C*_*i*_ = *C*_*j*_.

During our analysis, readers will see that the complexity of OC_0_ increases progressively.

### 2.1. Forms of van ‘t Hoff’s law adapted to diverse solute particle composition of solutions

We first take a closer look at how the diverse SP composition of a solution affects OC_0_ and thus osmotic pressure in a simple S-m-H_2_O with an ideal membrane:

(i) the original, simplest form of van ‘t Hoff’s law, *π* = *C·RT*, describes that if T is constant, the osmotic pressure in a simple osmosis system (S-m-H_2_O) is proportional to the molar concentration (C) of *S* containing a single species of non-dissociable solute molecules such as glucose or inulin. In this case, **C_TSP_** = C and *C*_i_ = *C*_j_. Because the membrane is ideal, the TSP of the solution are 100% impermeant, and the value of OC_0_ equals the value of **C_TSP_**, and thus ***π*** = *RT·OC*_0_;
(ii) if the single solute is a dissociable molecule, such as NaCl, van ‘t Hoff’s law is typically expressed as *π* = *gC*·*RT* [21,22]. Some physiology textbooks specify that [conventional] osmolarity = gC [15]. Since gC is identical to **C_TSP_** and the value of OC_0_ equals the value of **C_TSP_** (the membrane is ideal), the general form of van ‘t Hoff’s law, *π* = *RT*·*OC*_0_, covers this scenario as well; and
(iii) if a solution contains a mix of solute molecules, such as glucose, NaCl and MgSO_4_, the osmotic pressure can be represented in two equivalent forms. First, using a molecule (j)-based expression that accounts for the dissociation of each molecule: *π* = *π*_glu_ + *π*_NaCl_ + *π*_MgSO4_ = (*C*_glu_+*g*_NaCl_*C*_NaCl_ + *g*_MgSO4_*C*_MgSO4_)·*RT* [23,24]. The term inside the parentheses can be generalized to ${\sum g}_{j}C_{j}$. Again, $\sum {g_{j}C}_{j}$ is identical to **C**_**TSP**_, which in turn has the same value as OC_0_, and thus *π* = *RT*.*OC*_0_. Second, using an SP (i)-based expression that directly sums the concentrations of individual particles: *π* = (*C*_glu_+*C*_Na_ + *C*_Cl_+*C*_Mg_ + *C*_SO4_) ·*RT* = ($\sum C_{i}$)·*RT*, where ($\sum C_{i}$) refers to OC_0_.

### 2.2. Forms of van ‘t Hoff’s law adapted to both diverse solute particle composition and non-ideality of solutions

We are still considering a simple S-m-H_2_O with an ideal water-permeable membrane. When the solution is non-ideal, an osmotic coefficient (*Φ*) is used to account for its non-ideal behaviour [24–26]. Osmotic coefficients are specific for each type of solute molecule (not dissociated SP species) and can be determined experimentally by measuring how much a colligative property, such as freezing point depression, deviates from the ideal, expected value [25]. For electrolytes, the osmotic coefficients can be related directly to the activity coefficients of the solutes via a Debye–Hückel formulation and use of the Gibbs–Duhem relation [27]. Since the non-ideality of a solution affects its colligative properties, it thus affects a solution’s **C_TSP_**.

For example, if a solution contains a mix of non-dissociable and dissociable types of molecules such as glucose, NaCl and MgSO_4_, the non-ideality of the solution will affect the effective particle concentrations of all types of molecules (j) present in the solution: *π* = (*Φ*_glu_⋅*C*_glu_)⋅*RT*+*g*_NaCl_⋅(*Φ*_NaCl_⋅*C*_NaCl_)⋅*RT* + g_MgSO4_⋅(*Φ*_MgSO4_⋅*C*_MgSO4_)⋅*RT* = (Σ*g*_j_⋅(*Φ*_j_⋅*C*_j_)⋅*RT* [23], where Σg_j_⋅(*Φ*_j_⋅*C*_j_) is **C_TSP_**. Since the membrane is only permeable to water, the value of OC_0_ equals the value of **C_TSP_**, and thus *π* = *RT*⋅OC_0_.

### 2.3. Forms of van ‘t Hoff’s law adapted to solute particle-permeable membranes

A membrane’s permeability to a given SP species i (non-dissociable molecule particles or a dissociated ion species) is given by its reflection coefficient, *σ*_*i*_. If *σ*_*i*_ = 1, the membrane is impermeable to the particle i; if σ_i_ = 0, the membrane is freely permeable to i [15]:

(i) van ‘t Hoff’s law for a solution containing a single type of non-dissociable solute molecule (such as glucose) is presented as follows: *π* = *σC·RT* [28], where *σC* denotes the effective concentration of osmotically effective molecule particles, it reflects OC_0_ and hence *π = RT·OC*_0_;
(ii) if a solution contains one type of dissociable molecule, such as NaCl, van ‘t Hoff’s law is expressed as *π* = *σ·(g·C)·RT,* where σ applies to the NaCl molecule as a whole [15]. A more accurate expression may be *π* = (*σ*_Na_*C*_Na_ + *σ*_Cl_*C*_Cl_)*RT*, i.e. *π* = $\left(\sum \sigma_{i}C_{i}\right)$*RT*, because the membrane’s permeabilities for Na^+^ and Cl^-^ can be different. Yet this modification may not make much of a difference in reality because ions cannot cross the membrane as freely as non-charged particles. If we consider a membrane that has many channels open for Na^+^ but none for Cl^−^, *σ*_Cl_ = 1 >> *σ*_Na_, however, the initial movement of Na^+^ across the membrane will rapidly lead to the development of a membrane potential, which will effectively inhibit the further movement of Na^+^. In either case, *σ*·(*g·C*) (method 1) or $\left(\sum \sigma_{i}C_{i}\right)$ (method 2) represents the ‘osmotically effective fraction’ of **C_TSP_**, i.e. OC_0_, and therefore *π* = *RT·OC*_0_; and
(iii) if a solution contains a mix of non-dissociable and dissociable molecules (glucose + NaCl + MgSO_4_), applying method 1 or method 2 above, van ‘t Hoff’s law may be expressed in two ways:
  — method 1: applying σ_j_ to molecules: *π* = *π*_glu_ + *π*_NaCl_ + *π*_MgSO4_ = (*σ*_glu_*C*_glu_ + *g*_NaCl_*σ*_NaCl_*C*_NaCl_ + *g*_MgSO4_*σ*_MgSO4_*C*_MgSO4_)·*RT* = $\left(\sum g_{j}\sigma_{j}C_{j}\right)RT$; and
  — method 2: applying σ_i_ to particles: *π* = *π*_glu_ + *π*_NaCl_ + *π*_MgSO4_ = (*σ*_glu_*C*_glu_ + *σ*_Na_*C*_Na_ + *σ*_Cl_*C*_Cl_ + *σ*_Mg_*C*_Mg_ + *σ*_SO4_*C*_SO4_)·RT = $\left(\sum \sigma_{i}C_{i}\right)RT$.

However, in order to apply methods 1 and 2 to the solution, it must meet the condition that the given membrane is exclusively impermeable to either all cations or all anions. If this condition is not met, e.g. the membrane is permeable to Cl^−^ and Mg^2+^ but impermeable to Na^+^ and SO_4_^2−^, then Cl^−^ and Mg^2+^ will diffuse across the membrane and keep both compartments electrically neutral.

### 2.4. Forms of van ‘t Hoff’s law adapted to diverse solute particle composition of solutions, non-ideality of solutions and solute particle-permeable membranes

To be able to take these three aspects into account together, it can be reasoned that method 1 above is better suited to incorporate the three aspects into van ‘t Hoff’s law: *π* = *π*_glu_ + *π*_NaCl_ + *π*_MgSO**4**_ = (*σ*_glu_*Φ*_glu_*C*_glu_ + *g*_NaCl_*σ*_NaCl_*Φ*_NaCl_*C*_NaCl_ + *g*_MgSO4_*σ*_MgSO4_*Φ*_MgSO4_*C*_MgSO4_)⋅*RT* = $\left(\sum g_{j}\sigma_{j}\Phi_{j}C_{j}\right)RT\;$= $RT\cdot OC_{0}$.

Table 1 summarizes the multiple forms of van ‘t Hoff’s law. The text before RT in each form in the first column represents OC_o_. Hence, all of these can be replaced by a single general form of the law, *π* = RT⋅OC_0_. The third column of table 1 indicates how the key quantity **C_TSP_** is calculated in each of these scenarios.

**Table 1.** Multiple forms of van ‘t Hoff’s law adapted to diverse SP composition of solutions, non-ideality of solutions and SP-permeable membranes (not exclusive). (Table modified from [12] with permission from *The FASEB Journal*.)

form of van ‘t Hoff’s Law	application	calculation of C_TSP_	example solutes
***π*** *=* ***C****⋅RT*	single type of non-dissociable solute molecules; ideal solution; ideal membrane	C	glucose or albumin
***π*** *=* ***gC****⋅RT*	single type of dissociable solute molecules; ideal solution; ideal membrane	gC	NaCl or CaCl_2_
***π*** *=* ($\sum g_{j}C_{j}$)⋅RT	multiple types of solute molecules, non-dissociable and dissociable; ideal solution; ideal membrane	$\sum g_{j}C_{j}$	a mix of glucose, NaCl and MgSO_4_
***π*** *=* ($\sum g_{j}{Ф}_{j}C_{j}$)⋅RT	multiple types of solute molecules, non-dissociable and dissociable; non-ideal solution; ideal membrane	$\sum g_{j}\Phi_{j}C_{j}\;$	a mix of glucose, NaCl and MgSO_4,_
***π*** *=* ***σC****⋅RT*	single type of non-dissociable solute molecules; ideal solution; non-ideal membrane (permeable to water and some solute particles)	C	glucose or albumin
***π*** *=* ***σgC****⋅RT*	single type of dissociable solute molecules; ideal solution; non-ideal membrane	gC	NaCl or MgSO_4_
***π*** *= (*$\sum g_{j}\sigma_{j}C_{j}$*)⋅RT or* ***π*** *= (*$\sum \sigma_{i}C_{i}$*)⋅RT*	multiple types of solute molecules, non-dissociable and dissociable; ideal solution; non-ideal membrane	$\sum g_{j}C_{j}\;or\;\sum C_{i}$	a mix of glucose, NaCl and MgSO_4_
***π*** *= (*$\sum g_{j}\sigma_{j}{Ф}_{j}C_{j}$*)⋅RT*	multiple types of solute molecules, non-dissociable and dissociable; non-ideal solution; non-ideal membrane	$\sum g_{j}\Phi_{j}C_{j}\;$	a mix of glucose, NaCl and MgSO_4_

In the general form of van ‘t Hoff’s law, OC_0_ is now an umbrella term that encompasses the (complex) properties of the solution and the (complex) properties of the membrane that affect the magnitude of *π* in any given scenario:

(i) the diverse composition of a solution is reflected in *C*_i_ or *C*_j_ and *g*, and the use of the summation symbol, Σ;
(ii) the non-ideality of a solution factors into the value of OC_0_ through the osmotic coefficient, Φ. Different molecules have different values of Φ; and
(iii) the membrane’s permeability to solutes affects the value of OC_0_ via the reflection coefficient σ. Different SP species have different values of σ.

Even without the above analysis, the unified, general form of van ‘t Hoff’s law (*π* = *RT⋅OC*_0_) stands by itself logically: As long as T is kept constant, the magnitude of *π* is only proportional to OC_0_, the initial osmotically effective fraction of **C_TSP_** of the initial solution in the S compartment of S-m-H_2_O. The reasoning above demonstrates the appropriateness of the definition of OC_0_, the effectiveness of its application and its extreme complexity.

It should be noted that the content in table 1 applies if any one of the following criteria is met in S-m-H_2_O:

(i) the membrane is ideal;
(ii) the membrane is SP-permeable, but its permeability to the permeant SP is very low and thus negligible; and
(iii) the membrane is SP-permeable, but the size of the concentration gradient of the permeant SP is very small.

If the membrane’s permeability to any SP species is significant or not negligible, then the osmosis system is no longer a simple S-m-H_2_O. It should be considered a *complex* system that can be deconstructed into a simple osmosis system of the impermeant SP and a diffusion system of the permeant SP. For example, if a solution has a mix of urea and inulin (inu) and the given membrane is impermeable to inulin but relatively highly permeable to urea, then it can be viewed as the overlap of a simple osmosis system with an inulin solution (S_inu_-m_H2O_-H_2_O) and a urea diffusion system (S_urea_-m_urea_-H_2_O): S-m-H2O = S_inu_-m_H2O_-H_2_O + S_urea_-m_urea_-H_2_O, where m_H2O_ means the given m is H_2_O-permeable for the inulin osmosis system and m_urea_ means that the same m is urea-permeable for the urea diffusion system [29].

### 2.5. Limitations of the unified general form

The above approach to understand and deconstruct the factors that influence OC_0_ is still limited for several reasons:

(i) the value of the osmotic coefficient Φ of a solution with one type of molecule is dependent on the concentration of the solute [30,31], which adds complexity to OC_0_;
(ii) when dealing with the non-ideality of a solution with a mixture of different types of molecules (such as a solution with glucose, NaCl and MgSO_4_), the interaction among the multiple types of molecules or their dissociated cations and anions was not taken into consideration, which would add to the complexity of OC_0_;
(iii) for electrolyte solutions, the non-ideality of the solution (the osmotic and activity coefficients) will depend on the total ionic strength;
(iv) van ‘t Hoff’s law only applies to ideal and very dilute solutions. This means that the discussion of the complexity of OC_0_ is also limited in dilute solutions or probably at the boundary between dilute and less dilute solutions, which requires validation by simulation or experiments; and
(v) while most solutes in physiological fluids, such as electrolytes and glucose, can be effectively considered ‘dilute’ for our unified form, plasma proteins are a critical exception. Despite their low molar concentration, proteins’ large molecular size and strong intermolecular interactions (e.g. electrostatic forces and hydration effects) produce nonlinear relationships between oncotic pressure and concentration, typically modelled by specialized approaches such as the Landis–Pappenheimer formulation, virial expansions, or Donnan equilibrium models [32–34].

### 2.6. A more generalized form of van 't Hoff’s law

Owing to the inherent complexity of OC_0_, particularly in systems involving multiple solute species with differing reflection coefficients (*σᵢ*) and osmotic coefficients (*Φ*_*j*_), a more generalized form (extended general form) of van ’t Hoff’s law may be considered: *π*=θ⋅**C**_**TSP**_⋅*RT*. In this equation, **C**_**TSP**_ (concentration of TSP) serves as the independent variable, while θ is introduced as a system-level empirical coefficient, which we term the *osmosis system coefficient*. The product of θ·**C**_**TSP**_ replaces OC_0_ and captures a range of non-ideal behaviours and interactions that arise in complex osmosis systems, such as the combined influence of heterogeneous solutes, unequal membrane permeabilities and solute–solute or solute–membrane interactions, which OC_0_ alone may not adequately represent. Notably, *π* = *RT·OC*_0_ is actually a simplified form of *π* = θ⋅**C**_**TSP**_⋅*RT*, valid when θ·**C**_**TSP**_ reduces to OC_0_ under approximate or idealized conditions.

Importantly, θ is not derivable from first principles. Rather, it must be determined experimentally by measuring the osmotic pressure *π* and solving for θ using the equation *π* = θ⋅**C**_**TSP**_**⋅***RT*, or resolving from measured osmolarity (corrected osmolar activity) using an osmometer, i.e. θ = corrected osmolar activity/**C**_**TSP**_. Each osmosis system, defined by its particular solute composition, membrane properties and transport dynamics, has its own characteristic θ value. In this regard, θ is analogous to the single nephron filtration coefficient (SNKf) in renal physiology, widely used in the following relationship [35]: SNGFR = SNKf⋅Net Filtration Pressure, where SNKf reflects an empirically determined integration of membrane surface area, hydraulic conductivity and structural integrity—quantities that cannot be calculated from structural parameters alone. Similarly, θ captures the emergent behaviour of an osmosis system as a whole and must be derived from the measurement of *π* or osmolar activity rather than theory.

In brief:

(i) if OC_0_ can be reliably estimated from multiple *σ*ᵢ and *Φ*_*j*_, then the unified form *π* = *RT·OC*_0_ remains valid; and
(ii) if not, then the extended general form *π* = θ·**C**_**TSP**_·*RT* provides a practical, system-specific alternative grounded in experimental observation.

This dual-framework approach significantly extends the applicability of van ’t Hoff’s law to a broader range of real-world osmosis systems.

### 2.7. Initial validation of the general forms of van 't Hoff’s law

Since the comprehensive factors encapsulated by OC_0_ (*g, Φ, σ, Σ*) were derived theoretically, their ultimate applicability to *π* = *RT·OC*_0_ requires rigorous experimental validation in future studies. As θ can only be obtained through experimental measurement of osmotic pressure or osmolar activity, the extended general form, *π* = θ⋅**C**_**TSP**_⋅RT, must be validated by individual experiments for each combination of membrane, cell and solute. Nonetheless, to illustrate, we present two examples as initial validations using established osmotic pressure and osmolar activity data taken from the literature:

(i) validating *π* = *RT·OC*_0_ by using measured osmotic pressures exerted by dilute solutions with a single type of solute: in table 1 of Minkov *et al*.’s 2013 paper [36], the authors compared their measured osmotic pressures of sucrose solutions at increasing concentrations (generally dilute) at 22°C and at variable (open) and constant (closed) volume. Predicted osmotic pressures were also calculated using the original form of van 't Hoff’s law (*π*_Pred_ in our table 2, column 2). These authors’ measures of osmotic pressure at constant volume are selected for our initial validation (*π*_Exp_, see our table 2, column 3), as these values do not involve dilution of the solution as in the variable-volume measurements. Table 2 indicates that the unified form, *π* = *RT*·*OC*_0_, achieves complete reconciliation between their theoretical prediction and experimental observation. This validates OC_0_’s function as a system-level parameter that intrinsically corrects for thermodynamic non-ideality; and
(ii) validating *π* = θ·**C**_**TSP**_·*RT* by using the related data of physiological body fluids with a mix of approximately 20 different types of solutes in table 25-2 from *Guyton and Hall Textbook of Medical Physiology* [26]: table 3 shows the selected data from the textbook and how these data can be mapped to our new concepts of **C**_**TSP**_, θ·**C**_**TSP**_ and *π* = θ·**C**_**TSP**_·*RT*. Table 3 thus shows how our theoretical, extended general form can be applied to describe real world, physiological data, where θ·**C**_**TSP**_ inherently captures the collective non-ideal interactions among approximately 20 different solutes in table 25-2 of the textbook. These effects cannot be reproduced simply by applying individual osmotic coefficients or *OC*_0_ deducted from logical reasoning.

**Table 2.** Validation of *π* = RT·OC₀ using constant-volume osmotic pressure data for sucrose solutions from Minkov *et al*.’s 2013 paper [36] at 22°C. (Key parameters: σ = 1 (membrane impermeable to sucrose); *R* = 0.08314 L·bar/mol·K; *T* = 295.15 K; *RT* = 24.54 L·bar/mol; *C* = **C**_**TSP**_ (sucrose non-dissociable). Column definitions: (i) sucrose molar concentration; (ii) *π*_Theo_ = *RT·C* (uncorrected van 't Hoff prediction); (iii) *π*_Exp_ (experimental osmotic pressure from Minkov *et al*.’s table 1, column 3); (iv) error = *π*_Exp_ - *π*_Theo_ (negative values indicate systematic overprediction); (v) *Φ* = *π*_EXP_/(*RT·C*) (osmotic coefficient quantifying non-ideality); (vi) OC₀ = *σ·Φ·C* (effective osmotic concentration, resolves the error because *π*_Exp_ = *RT*·OC_0_ *≠ RT·C* in each row.)

concentration (C, mol L^−1^)	*π*_Pred_ = *RT·C* (bar)	*π*_Exp_ (bar)	*error* *(π*_Exp_ *- π*_The*o*_ *)*	*Φ* (*π*_Exp_/*RT·C*)	OC_0_ = *σ·Φ·C* (Osm L^−1^)
0.050	1.227	1.10	−0.127	0.897	0.0449
0.100	2.454	2.29	−0.164	0.933	0.0933
0.106	2.601	2.57	−0.031	0.988	0.1047
0.150	3.681	3.51	−0.171	0.954	0.1431
0.200	4.908	4.77	−0.138	0.972	0.1944
0.300	7.362	6.26	−1.102	0.850	0.2550

**Table 3.** Calculated osmotic pressures based on total solute particles (**C**_**TSP**_) and corrected osmolar activity from *Guyton and Hall Textbook of Medical Physiology*, table 25-2 [26]. (The ‘total osmotic pressure’ values in their table 25-2 were not used in our calculations. In their table, those values were obtained as corrected osmolar activity × 19.3 mmHg mOsm^−1^. In this study, osmotic pressures were computed using R = 62.3637 L·mmHg·mol⁻¹·K⁻¹ and T = 310.15 K (RT/1000 = 19.3421 mmHg mOsm^−1^), approximately 0.22% higher than 19.3 mmHg mOsm^−1^, leading to values approximately 12−13 mmHg greater than those in their textbook. This approach provides slightly more accurate estimates by using the exact gas constant and absolute temperature at 37°C.)

parameter	plasma (mOsm L^−1^)	interstitial fluid (mOsm L^−1^)	intracellular fluid (mOsm L^−1^)	mapping to concept in the present article
**total osmolarity of body fluid (mOsm L^−1^) with mixed solutes** (from table 25-2 of the textbook)	299.8	300.8	301.2	**C**_**TSP**_ = $\sum {g_{j}C}_{j}$ (theoretical value)
**corrected osmolar activity (mOsm L^−1^, experimental measurement) including the non-ideality of solution** (from table 25-2 of the textbook)	282.0	281.0	281.0	equivalent to θ·**C**_**TSP**_
**θ** (our calculation)	**0.9406**	**0.9342**	**0.9330**	$\theta =\frac{corrected\;osmolar\;activity}{C_{TSP}}$
**predicted osmotic pressure (π_Pre_**, **mmHg) without considering the non-ideality of the solution** (our calculation)	5799	5818	5824	π_Pre_ = **C**_**TSP**_⋅RT
**corrected osmotic pressure (π_corr,_ mmHg) calculated from corrected osmolar activity** (our calculation)	5454	5435	5435	π_corr_ = θ·**C**_**TSP**_·RT
**absolute error (π_Pre_ - π_corr,_ mmHg**)	345	383	389	covered by **θ**
**relative error (%**)	5.95%	6.58%	6.68%	covered by **θ**

Body fluids are dilute solutions. However, significant deviations between ideal prediction (*π*_Pred_) and activity-corrected osmotic pressures (*π*_Corr_) confirm non-ideality (5.95%–6.68% errors). Applying an osmosis system coefficient θ ≈ 0.93−0.94 fully resolves these errors, demonstrating its role in quantifying system-specific behaviour. The difference in the value of θ in the plasma, interstitial fluid and intracellular fluid highlights the nature of θ as a feature of the total osmotic system (an *emergent system property*, not a property of individual solutes).

While the original OC_0_-based general form is not intended for protein-containing solutions, the present use of the extended form in plasma (containing proteins) may be considered an exploratory test of its potential applicability to dilute solutions with proteins. Whether the extended form can reliably describe protein-containing fluids (such as the plasma) remains to be established by further experimental validation. The complexity of such systems may raise the possibility of nonlinear dependence of θ on parameters such as **C**_**TSP**_. This dependence would also have to be verified by experiment.

The two validation examples show that the central variable (*OC*_0_ or θ·**C**_**TSP**_) can account for experimental results already published, albeit without formal theoretical integration. They also show the explanatory power of these central variables. To further validate our work, more experiments using a range of solutes, membranes, concentrations and other variables need to be conducted in future studies.

### 2.8. Unified form of van ’t Hoff’s law in a composite osmosis system

**Figure 1.**
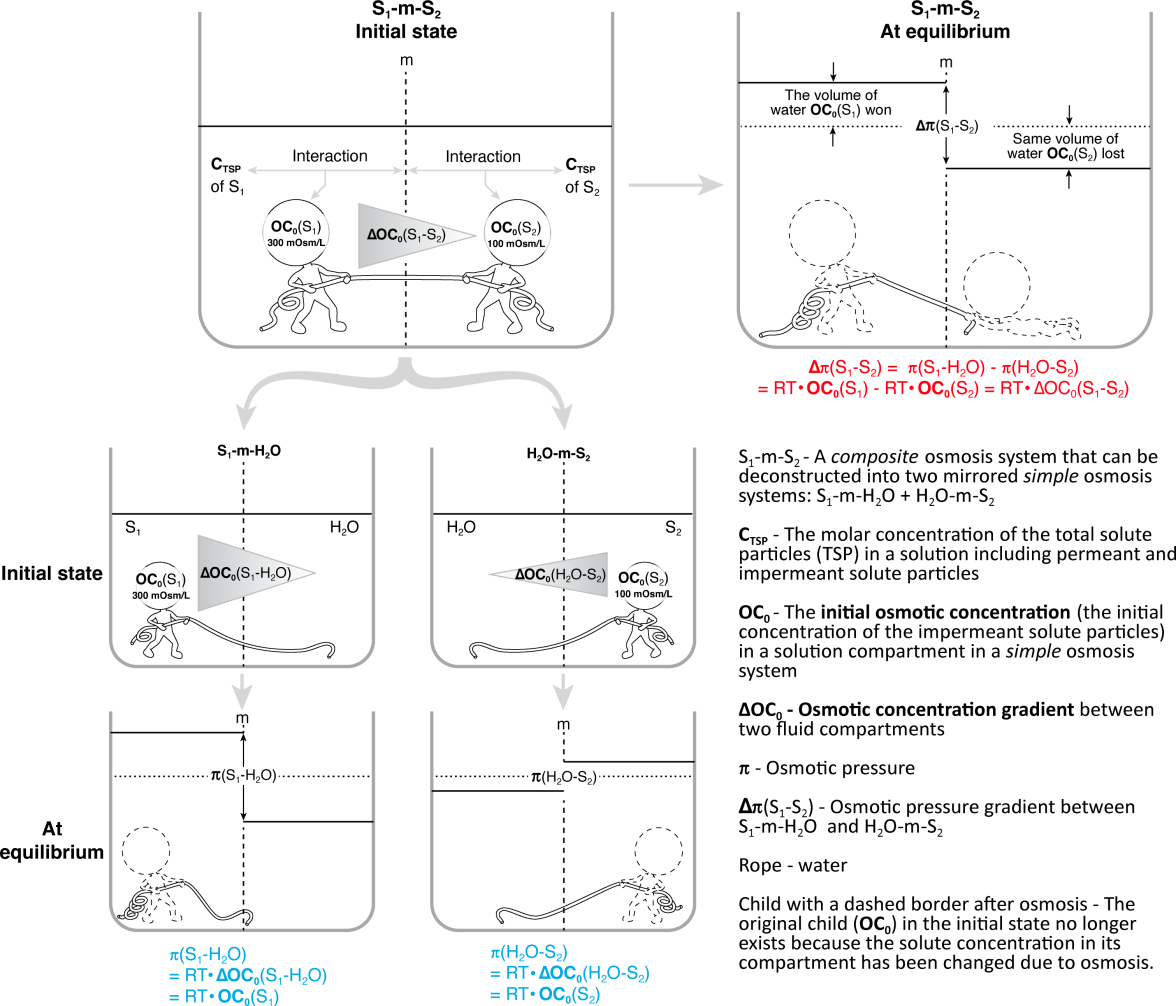
van ‘t Hoff’s law in a composite osmosis system. Osmosis in the composite osmosis system is compared to a competition for water between OC_0_(*S*_1_) and OC_0_(*S*_2_) as shown in the upper part. The system is also deconstructed into two mirrored simple osmosis systems (the lower-left part), with the general form of van ‘t Hoff’s law presented at the bottom of each simple osmosis system in blue. The resulting osmotic pressure gradient (Δ*π*) after osmosis stops is the sum of these two van ‘t Hoff’s law equations specified in red. Without the deconstruction, the weaker force driving water to the S_2_ compartment remains hidden or unrecognized. Image modified from [12], with permission from *The FASEB Journal*.

Either *π* = *RT*·OC_0_ or *π* = θ·**C**_**TSP**_·*RT* applies to a simple osmosis system. As introduced earlier, a composite osmosis system (S_1_-m-S_2_) can be deconstructed into two simple osmosis systems that are mirrored (S_1_-m-H_2_O and H_2_O-m-S_2_). Applying the unified form to a composite osmosis system, the formulations are shown below:

— *Δπ = RT·ΔOC*_*0*_ *(**figure 1**); or*
— *Δπ = θ·Δ* **C**_**TSP**_ *·RT*

### 2.9. Additional considerations and limitations of this article

First, the permeabilities of selectively permeable membranes to pure water vary [37], which may not affect the value of OC_0_ and the magnitude of *π* but will affect the duration of osmosis. This situation is not considered in this article, but it is of crucial importance for the design of osmotically controlled drug delivery systems (i.e. ‘osmotic pumps’), which deliberately manipulate the rate of drug release into the human body [38,39].

Second, although van ‘t Hoff’s law is widely used to estimate osmotic pressure, there remains disagreement regarding how to model osmosis quantitatively at the system level, particularly under non-ideal conditions [40–42]. Experimental evidence indicates that (osmotic) water flux across a selectively permeable membrane is two to six times faster than water diffusion [43,44]. These findings suggest that water transport during osmosis does not occur at the rate of diffusion [20,45].

Third, the idea that the solute dilutes the solvent (water) and thus establishes a gradient in water concentration which drives osmosis has been challenged as well [20]. ‘Perhaps the most persuasive of these [arguments] is the fact that the addition of solute does not necessarily dilute water. Many aqueous salts instead have a concentrating effect, because the electric charge of their ions disrupts the relative open hydrogen bond network of liquid water’ [20].

Fourth, as mentioned in §1, a microscopic explanation for the generation of the osmotic force has been available in the field of physics [19,20,46,47]. Kinetic models of osmotic motion, for example, can account for the temperature-dependence of osmotic flow and the influence of reflection coefficients on osmotic pressures [48]. However, even after several decades, this information has not widely penetrated biomedical disciplines, and many textbooks in biomedical fields still teach that osmosis is water diffusion across a selectively permeable membrane, indicating a gap between microscopic and macroscopic studies.

Fifth, how the permeant fraction of **C_TSP_** affects osmosis and OC_0_ has been completely ignored in current textbooks and also in this article so far. The permeant fraction is not merely osmotically ineffective but may exert an anti-osmosis effect during osmosis.

Finally, while the present study is theoretical in nature, as mentioned at the end of §2.7, we acknowledge the need for empirical validation in the future. In addition, while θ serves as an empirical system-level coefficient, future work may also explore its variability and sensitivity across different systems through parameter analysis, which may help further characterize the complexity of non-ideal osmotic behaviour. We also hope future studies—particularly those with access to simulation tools or experimental facilities—will further validate and refine this unifying approach.

## 3. Conclusion

First, by introducing the concept of OC_0_, the initial OC in the S compartment of a simple osmosis system (S-m-H₂O), as a system-level parameter (rather than a property of the solution itself), we demonstrate that OC_0_ integrates multiple contributing factors, particularly the reflection coefficients of different SP species (σᵢ) and the osmotic coefficients of various solute molecules (*Φ*_*j*_). OC_0_ naturally unifies the diverse existing forms of van ’t Hoff’s law into a single general form: *π* = *RT·OC*_0_. This unification demonstrates the appropriateness, usefulness and effectiveness of OC_0_.

Second, the mathematical derivation provided in this article illustrates the complexity of OC_0_ and how it reflects system-specific interactions.

Third, in practice, the interactions among SP, solvent molecules (H₂O) and the membrane can be even more complex, sometimes exceeding what can be fully captured by *σ*_*i*_*, Φ*_*j*_*,* or OC_0_ alone. To address such cases, we propose a more generalized form (extended general form): *π* = θ·**C**_**TSP**_·*RT*, where **C**_**TSP**_ denotes the molar concentration of TSP in the S compartment, and θ is the osmosis system coefficient, an empirical parameter introduced to account for complexities that OC_0_ cannot fully represent.

Unlike OC_0_, θ cannot be theoretically derived from first principles; rather, it must be determined experimentally by fitting the equation *π* = θ·**C**_**TSP**_*·RT* to measured osmotic pressure data. The previously defined form, *π* = *RT·OC*_0_, can be viewed as a special, simplified case of this form, in which θ·**C**_**TSP**_ reduces to OC_0_.

Fourth, the introduction and application of the two fundamental osmosis systems and the concepts of **C**_**TSP**_ and OC_0_ fill a longstanding theoretical gap in the macroscopic understanding of osmosis, one that has persisted for over a century since the original formulation of van ’t Hoff’s law. Building on this foundation, the two generalized forms of van ’t Hoff’s law proposed in this study represent a further theoretical advancement, offering a practical and versatile framework for both scientific research and education.

The theoretical and practical significance of this work for the macroscopic study of osmosis is self-evident. While these two general forms represent an important theoretical step forward, they are nonetheless applicable only to dilute solutions.

Finally, a deeper investigation into the complexity of OC_0_ and θ·**C**_**TSP**_ will require interdisciplinary collaboration, particularly among researchers in chemistry, physics and biomedical areas.

## Data Availability

All data supporting this study, including detailed mathematical derivations and logical reasoning steps, are included in the article. No additional data are available.

## Footnotes

^1^
Not many textbooks or articles specify what osmotic concentration is in a given form of van ‘t Hoff’s law. What was specified in the textbook is actually **C**_**TSP**_ [15] implicitly facing an ideal membrane, so that the value of OC_0_ = value of **C**_**TSP**_.
